# Characteristics Associated with Good Self-Perceived Mental Health among United States Adults with Arthritis

**DOI:** 10.3390/bs12080256

**Published:** 2022-07-27

**Authors:** Nouf Bin Awad, David R. Axon

**Affiliations:** Department of Pharmacy Practice & Science, R. Ken Coit College of Pharmacy, The University of Arizona, Tucson, AZ 85721, USA; nbawad@pharmacy.arizona.edu

**Keywords:** arthritis, autoimmune diseases, mental health, national survey

## Abstract

Mental health disorders are prevalent among United States (US) adults with arthritis. Yet, little is known about characteristics associated with mental health among US adults with arthritis. This retrospective cross-sectional study used 2019 Medical Expenditures Panel Survey data to assess the association between multiple personal characteristics and mental health status among US adults with arthritis. Hierarchical logistic regression models modeled associations between personal characteristics and mental health status. Model 1 included predisposing factors, model 2 included predisposing and enabling factors, while model 3 included predisposing, enabling, and need factors. The a priori alpha level was 0.05. Analyses accounted for the complex survey design and were weighted to produce national estimates. Among 28,512 individuals, 4984 met the inclusion criteria. Of these, 4181 had good mental health (85.5%, 95% confidence interval (CI) = 84.3%, 86.7%). The following characteristics were associated with good mental health status in the final adjusted model: age 18–64 vs. ≥65 (adjusted odds ratio (AOR) = 0.29, 95% CI = 0.12, 0.71), Midwest vs. West census region (AOR = 5.17, 95% CI = 1.63, 16.46), no degree vs. higher than high school education (AOR = 0.34, 95% CI = 0.12, 0.92), and high school diploma vs. higher than high school education (AOR = 0.40, 95% CI = 0.18, 0.86). In conclusion, this study suggests such characteristics may be targeted to help improve mental health among this population. Additional efforts are needed to help satisfy the unmet need for mental healthcare among this population.

## 1. Introduction

Having an autoimmune condition, such as arthritis, can lead to the development of psychological stress and mental health disorders [1]. Arthritis is a prevalent condition affecting 58.5 million United States (US) adults in 2021 [2]. There are several types of arthritis, including osteoarthritis, rheumatoid arthritis, and psoriatic arthritis [3]. The most common type of arthritis is osteoarthritis, which entails wear and tear in joints due to overuse [4,5]. Symptoms of osteoarthritis include pain, swelling, and tenderness [6], while symptoms of rheumatoid arthritis include deep joint aching, trouble dressing, bending, squatting, gripping things, pain while walking, and stiffness [4].

Individuals with arthritis may suffer from mental health issues because of the interaction of arthritis with other health conditions and their functional states such as disability, pain, and fatigue [7]. Individuals with arthritis also experience additional challenges, such as medication side effects, less physical activity, and poorer quality of life. This in turn can lead to mental health conditions such as anxiety and depression, poorer social functioning, and greater pain intensity. The prevalence of depression among individuals with arthritis varies based on the kind of arthritis, the instruments, and thresholds used to diagnose depression, the country of residency, and the definition of depression [8]. Depression has been associated with poor health-related quality of life (HRQOL), disability, death, significant financial burden, other arthritis-related problems, and pain [8]. In addition, arthritis can have a negative impact on an individual’s HRQOL [9]. HRQOL is an important outcome when managing chronic conditions such as arthritis. HRQOL can be influenced by several dimensions, including the physical dimension (pain and deterioration of physical functioning), the psychological dimension (anxiety and depression), the cognitive dimension (memory and attention), and the social dimension (self-esteem and interpersonal relationships) [10]. Several studies have found factors such as fatigue, pain intensity, stiffness, and functional limitations can affect the quality of life among individuals with arthritis [11,12,13]. Furthermore, socio-economic factors such as age, lifestyle habits, employment, economic status, and comorbidities have also been shown to affect quality of life [9,13,14].

If an individual with arthritis experiences a deterioration in one aspect of their health, it can directly or indirectly affect other areas of their health [7]. In addition, individuals with arthritis incur high health costs and healthcare service utilization. For example, one cross-sectional study that used 2011 Medical Expenditure Panel Survey (MEPS) data reported the cost of hospitalization due to poor physical and mental health for individuals with rheumatic conditions was between USD 10,000 and USD 50,000 in 2011 [15].

When compared to the general population, the prevalence of mental health issues is higher among people with arthritis [7,16,17,18]. For instance, one cross-sectional study that used 2002 National Health Interview Survey (NHIS) data reported the prevalence of serious psychological distress and frequent anxiety or depression was significantly higher in adults with arthritis than in the general population [7]. Another study analyzed data from the National Comorbidity Survey Replication and found that 27.3% of 9282 respondents had arthritis in 2001–2003, of which 24.3% also reported having a mental disorder in the past 12 months [16]. Another study using 2004–2006 MEPS data found that most rheumatoid arthritis patients experienced depression [19], while one further study using 2003 MEPS data found that most individuals with rheumatoid arthritis had mental health issues because of their symptoms [20]. A nationwide longitudinal study found a strong bidirectional relationship between arthritis and depression [20]. Additionally, a narrative review of the impact of mental health on patient-reported outcomes found that mental health issues may persist even if patients have lower levels of inflammation. Depression is twice as frequent in rheumatoid arthritis patients as it is in the general population, and cross-sectional studies have revealed a bidirectional association between mental health disorders and rheumatoid arthritis. Chronic inflammation inhibits physiological stress responses, including appropriate coping behaviors, leading to depression and poorer long-term prognosis among individuals with rheumatoid arthritis. The pain score among individuals with rheumatoid arthritis is not always entirely connected to inflammatory arthritis and immunological disease activity. For instance, non-inflammatory pain, anxiety, sleep disturbance, depression, psychosocial situation, fibromyalgia, mechanical pain, neuropathic pain, fatigue, low mood, and social well-being are all known to contribute to total pain [21,22,23,24].

Despite our existing knowledge, little is known about the factors that are associated with mental health status among US adults with arthritis. This information could be useful for target interventions that may improve health outcomes for these individuals. Thus, this retrospective cross-sectional study aimed to estimate the prevalence of arthritis and to determine the predisposing, enabling, and need factors associated with good mental health status among US adults with arthritis.

## 2. Methods

### 2.1. Study Design and Data Source

This was a retrospective cross-sectional study that used MEPS data from 2019. MEPS data are collected on behalf of the Agency for Healthcare Research and Quality (AHRQ) using the sampling framework from the NHIS. Data are collected for variables such as healthcare cost and utilization, income, insurance, demographics, health conditions, and health status in five rounds over a two-year period. Using the weighting variable provided by MEPS, nationally representative estimates of the civilian non-institutionalized US population can be calculated [25]. This study used the 2019 full-year consolidated data file as this is the most recent publicly available data at the time of this study, which contained data on 28,512 individuals [26,27]. The study was approved by the University of Arizona Institutional Review Board (IRB number: 2021-016-PHPR).

### 2.2. Study Participants

Study participants consisted of US adults (≥18 years) alive for the full 2019 calendar year with arthritis. Arthritis was identified using the MEPS variable “ARTHDX” which to asked participants if they had ever been told by a doctor or other health professional that they had arthritis [26,27].

### 2.3. Dependent Variable

The dependent variable in this study was self-perceived mental health state. This was determined based on responses to a question that asked people to describe their mental health as either excellent, very good, good, fair, or poor [26,27]. For the purpose of this study, dichotomization was performed in order to differentiate between respondents with good versus poor mental health. Good mental health included responses of excellent, very good, and good, while poor mental health included responses of fair and poor.

### 2.4. Independent Variables

Independent variables were categorized into one of three groups of factors (predisposing, enabling, need) using an adaptation of Andersen’s Behavioral Model [28]. First, predisposing factors contained age (18–64 years, ≥65 years); gender (male, female); race (white, other); ethnicity (Hispanic, non-Hispanic); census region (Northeast, Midwest, South, West). Second, enabling factors contained education status (less than high school, high school diploma or equivalent, higher than high school); employment status (employed, unemployed); marital status (married, other); poverty status (poor/near poor/low income, middle/high income). Third, need factors contained use of assistive devices (yes, no); difficulty walking/climbing stairs (yes, no); limitation in physical functioning (yes, no); joint pain (yes, no); the number of chronic conditions from the following list—high blood pressure, coronary heart disease, angina, heart attack, other heart diseases, stroke, emphysema, bronchitis, high cholesterol, cancer, diabetes, asthma (≥2 conditions, <2 conditions).

### 2.5. Data Analysis

Differences between study participants with good self-perceived mental health and poor self-perceived mental health were compared using chi-square tests. Hierarchical logistic regression models were employed to assess the associations between independent variables and self-perceived mental health state. Model 1 included the predisposing factors, model 2 included the predisposing and enabling factors, while model 3 (fully adjusted model) included the predisposing, enabling, and need factors. The analysis modeled the good self-perceived mental health state, with the poor self-perceived mental health state serving as the reference group. The a priori alpha level was 0.05. Cluster and strata variables were used to maintain the structure of the MEPS data, and the appropriate weighting variable (provided in the MEPS dataset) was used to obtain nationally representative estimates of the prevalence of good and poor mental health status among eligible civilians, non-institutionalized US adults with arthritis. All analyses were conducted using SAS OnDemand for academics, university edition (SAS institute Inc., Cary, NC, USA).

## 3. Results

**Figure 1:**
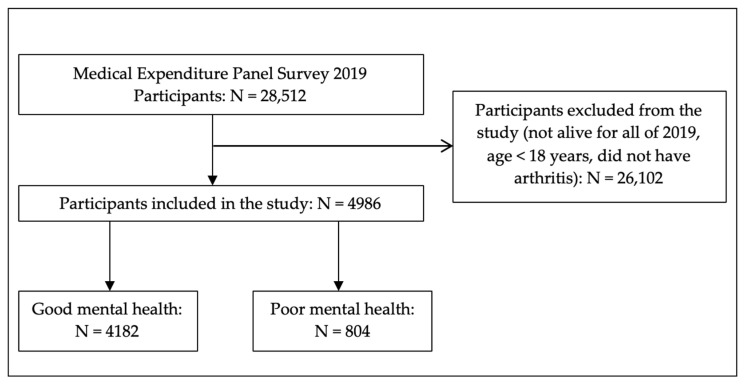
Number of participants included in the study.

The number of participants included in this study is shown in Figure 1. Out of 28,512 total participants in the 2019 full-year consolidated data file, 4986 were eligible for this study (good mental health = 4182, poor mental health = 804). The majority of participants reported good mental health (weighted study population = 51,399,277; 85.5%, 95% confidence interval (CI) = 84.3, 86.7) while the minority of participants reported poor mental health (weighted study population = 8,719,541; 14.5%, 95% CI = 13.3, 15.7).

The demographic characteristics of US adults (≥18 years) with arthritis stratified by good or poor self-perceived mental health state are shown in Table 1. There was a statistically significant difference between all variables (*p* < 0.05) except census region (*p* = 0.54) and joint pain (*p* = 0.14).

The factors associated with good self-perceived mental health among US adults (≥18 years) with arthritis are shown in Table 2. The fully adjusted model (model 3) indicated three variables had a statistically significant association with mental health status: age, census region, and education status. People aged 18–64 were less likely to report good mental health versus people aged ≥65 (adjusted odds ratio (AOR) = 0.317, 95% confidence interval (CI) = 0.138, 0.729). People in the Midwest census region were more likely to report good mental health than people in the West census region (AOR = 4.508, 95% CI = 1.493, 13.613). People who had completed less than high school education (AOR = 0.306, 95% CI = 0.108, 0.864) and people who had only completed high school education (AOR = 0.381, 95% CI = 0.173, 0.837) were less likely to report good mental health than people with higher than high school education.

## 4. Discussion

The key findings from this study were that the majority (85.5%) of US adults (≥18 years) with arthritis reported good mental health, and that age, census region, and education status were significantly associated with self-perceived mental health status among US adults (≥18 years) with arthritis. These findings are the focus of the discussion and are elaborated on below.

In our study, the weighted analysis indicated just over 60 million US adults (≥18 years) had arthritis in 2019. This finding is similar to the results of previous studies [29,30,31]. For instance, a study using NHIS data found that 52.5 million US adults had arthritis in 2010–2012 [29]. Another study using NHIS data found that 54.4 million US adults had arthritis in 2013–2015 [30]. A further study using NHIS data found that 61.1 million people aged 18 to 64 in the US were affected by arthritis in 2015 [31]. These findings also suggest that the prevalence of arthritis is increasing, as confirmed by the CDC, who predicted the prevalence of doctor-diagnosed arthritis is expected to increase from 58.5 million US adults in 2013–2015 to an estimated 78.4 million US adults by 2040 [29]. The increase in the prevalence of arthritis will also lead to an estimated 52% increase in arthritis-attributable activity limitations, reaching 34.6 million cases by 2040 [29]. These findings highlight the increasing burden of arthritis among US adults and the need for appropriate health policies and preventive care to help manage this condition.

Among the US adults who had arthritis in 2019, the majority (85.5%) reported having good mental health. This is an interesting finding given that previous studies have shown individuals with arthritis have poorer mental health compared to those without arthritis [8,17,32,33]. For example, a previous cross-sectional study in 17 countries found that mood and anxiety disorders were more common among individuals with arthritis than those without arthritis after adjustment for age and sex (OR = 1.9 for individuals with arthritis versus those without arthritis) [33]. There are several possible explanations for this difference, including different definitions of mental health status, and different countries or populations. For example, our study used a broad definition of mental health (rather than specific mental health conditions) and only included US adults with arthritis. Although our study showed that approximately 85% of US adults with arthritis had good self-reported mental health, this means that approximately 15% of US adults with arthritis had poor self-reported mental health. This is lower than the general adult population, where the prevalence of poor mental health is approximately 21% in 2020 [6] and approximately 20% in 2019 [34]. Recent studies indicate an increase in the proportion of US adults reporting poor mental health. For instance, one cross-sectional study of a national sample of 963 US adults conducted at the start of the COVID-19 pandemic (March 2020) found that mental health disorders were prevalent throughout the US, in particular among younger adults [35]. These findings suggest that additional efforts are required to help improve the mental health of US adults with arthritis. For instance, routine mental health screening with appropriate referral to mental health management programs and professionals may also be important to help address the burden of poor mental health among this population [17]. In addition, research has found that self-management education workshops and physical activity programs may help improve mental health in this population [36]. Encouraging individuals with arthritis to be physically active could help improve their mood and energy, as well as reduce symptoms of anxiety, depression, and arthritis [36,37]. Self-management education is an interactive community-based workshop that educates individuals on how to manage arthritis or other chronic diseases. Such programs include the Chronic Disease Self-Management Program (CDSMP); Tomando Control de su Salud; Better Choices Better Health^®^; Better Choices Better Health^®^ for Arthritis; Tool Kit for Active Living. In addition, the Arthritis Self-Management Program (ASMP) helps participants learn and experience the many approaches required to create a personalized self-management program and have the confidence to implement it [38]. Community-based, structured arthritis-appropriate, physical activity programs include Arthritis Foundation Aquatic Program (AFAP); Active Living Everyday (ALED); EnhanceFitness^®^ (EF); Fit & Strong; Walk with Ease (WWE); Arthritis Foundation Exercise Program (AFEP). These programs have been shown by the CDC to minimize arthritis symptoms and improve the quality of life for individuals with arthritis [37].

In our study, those aged 18–64 years were less likely to report good mental health than those aged ≥65 years (AOR = 0.317, 95% CI = 0.138, 0.729). Although there is limited literature on the association between age and mental health among US adults with arthritis, the Arthritis Conditions Health Effects Survey found nearly one-third of 1793 respondents with arthritis aged 45 years and older reported having anxiety (30.5%), depression (17.5%) or both (14.7%) in 2005–2006 [39]. Among the general population, a 2018 CDC report stated that adults aged 18 to 44 were more likely than older adults to experience symptoms of anxiety or depression [36]. Another study found adults aged 45 to 64 and adults aged ≥65 were less likely to seek mental health clinics compared to adults aged 18 to 44 [17]. This finding suggests the need for future work to investigate the role of age in the association between arthritis and mental health. A further study demonstrated that psychological well-being was associated with aging, with older adults progressively more satisfied with their mental health than younger adults [40]. These findings suggest that it may be beneficial to implement policies and interventions that promote positive mental health, particularly for younger adults with arthritis.

In our study, adults living in the Midwest census region were more likely to report good mental health than adults living in the West census region (AOR = 4.508, 95% CI = 1.493, 13.613). Although there are reports of the national frequency of mental health illness among adults with arthritis, [32,39] there is insufficient information regarding state-specific or regional prevalence, especially for common mental health disorders. One study using 2017 Behavioral Risk Factor Surveillance System (BRFSS) data found that frequent mental health issues and depression were commonly reported by adults with arthritis throughout the US, with particularly high prevalence in Appalachian and southern states [41]. The findings from our study and the limited existing literature suggests there is a need to further explore mental health among US adults with arthritis at a more local level, which may offer greater insight into areas where interventions may be best targeted to improve mental health among this population.

Adults in our study who had not completed high school education (AOR = 0.306, 95% CI = 0.108, 0.864) and adults who had completed high school education (AOR = 0.381, 95% CI = 0.173, 0.837) were less likely to report good mental health than adults with higher than high school education. Previous research has found less educated US adults with arthritis and serious psychological distress were less likely to seek mental health clinics compared to adults with arthritis only [31]. This difference may be due to the different populations studied, or that the likelihood of seeking mental health services may not be correlated with having a mental health issue—it may be that less educated people have less access to healthcare (e.g., poorer health insurance coverage, less money and time to attend clinics) than their better-educated counterparts. This information is important for future work to investigate the barriers to mental health help-seeking and to improve mental health access for low-income people. Finally, a systematic review and meta-analysis found that patient education may help people with rheumatoid arthritis improve their clinical outcomes and psychological well-being [42]. The findings from our study add support to the notion of providing educational intervention to help US adults with arthritis better manage their health conditions.

There were some limitations of this study. This was a retrospective study using secondary data that were collected for purposes other than this study. This study can only conclude a statistical association rather than a temporal relationship between the variables investigated. Data were self-reported by MEPS participants five times over a two-year period; thus there is a possibility of recall bias. In addition, this study did not include data on mental health outcomes, which would have been helpful to inform targeted interventions among this population. However, strengths of the study include the nationally representative sample of civilian, non-institutionalized US residents which offers good external validity to the findings.

## 5. Conclusions

In conclusion, this study found that the majority of US adults (≥18 years old) with arthritis reported having good mental health, and that age, census region, and education status were associated with reporting good mental health. These findings provide insight into the characteristics associated with mental health and may be suitable targets for interventions to improve mental health among US adults with arthritis. The findings of this study suggest that additional effort is needed to help improve the mental health of approximately 15% of the US adult population with arthritis. Early mental health screening with interventions or referrals to specialist providers may help improve health outcomes among this population.

## Figures and Tables

**Table 1:** Demographic characteristics of United States adults (≥18 years) with arthritis, stratified by good or poor self-perceived mental health state.

Characteristics	Good Mental Health N = 4182 Weighted Percent (95% CI)	Poor Mental Health N = 804 Weighted Percent (95% CI)	Total N = 4986 Weighted Percent (95% CI)	*p*-Value
Predisposing factors:				
Age				<0.0001
18–64 years	48.3 (46.0, 50.6)	58.7 (54.9, 62.5)	49.8 (47.7, 51.9)	
≥65 years	51.7 (49.4, 54.0)	41.2 (37.4, 45.1)	50.2 (48.1, 52.3)	
Gender				0.04
Male	39.8 (38.1, 41.5)	35.3 (31.3, 39.3)	39.2 (37.6, 40.8)	
Female	60.2 (58.5, 61.9)	64.7 (60.7, 68.7)	60.8 (59.2, 62.4)	
Race				0.05
White	81.9 (80.0, 83.7)	78.2 (74.3, 82.2)	81.3 (79.5, 83.1)	
Other	18.1 (16.3, 20.0)	21.8 (17.8, 25.7)	18.7 (16.9, 20.5)	
Ethnicity				<0.0001
Hispanic	8.0 (6.9, 9.2)	13.9 (10.1, 17.6)	8.9 (7.6, 10.2)	
Non-Hispanic	92.0 (90.8, 93.1)	86.1 (82.4, 89.9)	91.1 (89.8, 92.4)	
Census region				0.54
Northeast	17.2 (14.4, 20.1)	16.0 (11.8, 20.1)	17.1 (14.3, 19.8)	
Midwest	24.1 (21.7, 26.5)	22.0 (18.5, 25.4)	23.8 (21.6, 26.0)	
South	38.6 (35.7, 41.5)	41.7 (37.1, 46.2)	39.1 (36.4, 41.8)	
West	20.0 (17.8, 22.3)	20.4 (16.7, 24.0)	20.1 (18.0, 22.2)	
Enabling factors:				
Education status				<0.0001
Less than high school	9.5 (8.4, 10.6)	22.6 (19.2, 25.9)	11.4 (10.2, 12.6)	
High school diploma or equivalent	49.5 (47.4, 51.6)	52.1 (47.8, 56.3)	49.9 (48.0, 51.7)	
Higher than high school	41.0 (38.8,43.2)	25.4 (21.2, 29.5)	38.7 (36.8, 40.7)	
Employment status				<0.0001
Employed	44.4 (42.4, 46.4)	27.0 (23.0, 31.1)	41.9 (39.9, 43.8)	
Unemployed	55.6 (53.6, 57.6)	73.0 (68.9, 77.0)	58.1 (56.2, 60.1)	
Marital status				<0.0001
Married	56.8 (54.8,58.7)	40.2 (35.8, 44.6)	54.4 (52.6, 56.2)	
Other	43.2 (41.3,45.2)	59.8 (55.4, 64.2)	45.6 (43.8, 47.4)	
Poverty status				<0.0001
Poor/near poor/low income	26.8 (25.0, 28.5)	51.4 (46.7, 56.0)	30.3 (28.6, 32.1)	
Middle/high income	73.2 (71.5, 75.0)	48.6 (44.0, 53.3)	69.7 (67.9, 71.4)	
Need factors:				
Use of assistive devices				<0.0001
Yes	17.3 (15.9, 18.8)	38.3 (34.6, 42.0)	20.4 (19.0, 21.8)	
No	82.7 (81.2, 84.1)	61.7 (58.0, 65.4)	79.6 (78.2, 81.0)	
Difficulty walking/climbing stairs				<0.0001
Yes	24.7 (23.0, 26.3)	50.5 (45.9, 55.1)	28.4 (26.7, 30.1)	
No	75.3 (73.7, 77.0)	49.5 (44.9, 54.1)	71.6 (69.9, 73.3)	
Limitation in physical functioning				<0.0001
Yes	34.0 (32.0, 36.0)	62.1 (58.1, 66.1)	38.1 (36.3, 39.9)	
No	66.0 (64.0, 68.0)	37.9 (33.9, 41.9)	61.9 (60.1, 63.7)	
Joint pain				0.14
Yes	59.3 (54.2, 64.5)	71.2 (57.5, 85.0)	60.8 (55.9, 65.6)	
No	40.7 (35.5, 45.8)	28.8 (15.0, 42.5)	39.2 (34.4, 44.1)	
Chronic conditions				<0.0001
≥2 conditions	60.4 (58.6, 62.2)	72.4 (69.0, 75.9)	62.1 (60.6, 63.7)	
<2 conditions	39.6 (37.8, 41.4)	27.6 (24.1, 31.0)	37.9 (36.3, 39.4)	

Footnote: Analysis based on 4986 United States adults (≥18 years) alive during the 2019 calendar year with a diagnosis of arthritis in the 2019 Medical Expenditure Panel Survey (MEPS) full-year consolidated data file. Self-perceived mental health state was determined based on responses to a question that asked participants to rate their mental health status as: excellent, very good, good, fair, or poor. For the purpose of this study, good self-perceived mental health included the responses of excellent, very good, and good, while poor self-perceived mental health included the responses of fair and poor. Chi-square tests were used to assess differences between good and poor self-perceived mental health groups.

**Table 2:** Factors associated with good self-perceived mental health among United States adults (≥18 years) with arthritis.

Factors	Model 1 Odds Ratio (95% CI)	Model 2 Odds Ratio (95% CI)	Model 3 Odds Ratio (95% CI)
**Predisposing factors:**			
Age (18–64 vs. ≥65 years)	**0.680 (0.571,0.812)**	**0.461 (0.374, 0.569)**	**0.317 (0.138, 0.729)**
Gender (Male vs. female)	1.207 (0.998, 1.460)	1.026 (0.838, 1.256)	1.085 (0.537, 2.192)
Race (White vs. other)	1.245 (0.980, 1.582)	0.960 (0.751, 1.226)	0.389 (0.147, 1.027)
Ethnicity (Hispanic vs. non-Hispanic)	**0.549 (0.407, 0.740)**	0.747 (0.537, 1.040)	0.867 (0.368, 2.044)
Census region (Northeast vs. West)	1.055 (0.759, 1.466)	1.126 (0.799, 1.587)	1.473 (0.547, 3.966)
Census region (Midwest vs. West)	1.047 (0.789, 1.389)	1.109 (0.829, 1.484)	**4.508 (1.493, 13.613)**
Census region (South vs. West)	0.935 (0.720, 1.213)	1.042 (0.792, 1.371)	1.941 (0.784, 4.802)
**Enabling factors:**			
Education status (less than high school vs. higher than high school)		**0.482 (0.345, 0.673)**	**0.306 (0.108, 0.864)**
Education status (high school diploma or equivalent vs. higher than high school)		**0.758 (0.586, 0.981)**	**0.381 (0.173, 0.837)**
Employment status (employed vs. unemployed)		**2.311 (1.785, 2.991)**	1.301 (0.548, 3.090)
Marital status (married vs. other)		**1.524 (1.233, 1.884)**	1.627 (0.758, 3.493)
Poverty status (poor/near poor/low income vs. middle/high income)		**0.595 (0.69, 0.754)**	0.759 (0.348, 1.656)
**Need factors:**			
Use of assistive devices (Yes vs. no)			0.630 (0.235, 1.690)
Difficulty walking/climbing stairs (Yes vs. no)			0.918 (0.363, 2.326)
Limitation in physical functioning (Yes vs. no)			0.886 (0.320, 2.456)
Joint pain (No vs. yes)			1.637 (0.783, 3.423)
Chronic conditions (≥2 conditions vs. <2 conditions)			0.507 (0.235, 1.092)

Footnote: Analysis based on 4986 United States adults (≥18 years) alive during the 2019 calendar year with a diagnosis of arthritis in the 2019 Medical Expenditure Panel Survey (MEPS) full-year consolidated data file. Excellent/very good/good self-perceived mental health n = 4182; fair/poor self-perceived mental health n = 804 (reference group). Model 1 included predisposing factors (age, gender, race, ethnicity, census region), model 2 included predisposing and enabling factors (age, gender, race, ethnicity, census region, education status, employment status, marital status, poverty status), and model 3 included predisposing, enabling, and need factors (age, gender, race, ethnicity, census region, education status, employment status, marital status, poverty status, use of assistive devices, difficulty walking/climbing stairs, limitation in physical functioning, joint pain, number of chronic conditions). CI: confidence interval. Model 1 c-statistic: 0.604, Wald statistic <0.0001; model 2 c-statistic: 0.709, Wald statistic <0.0001; Model 3 c-statistic: 0.751, Wald statistic: 0.0002. Bold indicates that the value is statistically significant.

## Data Availability

Data are available from the corresponding author on request.
